# Development of a Critical Nitrogen Dilution Curve of Double Cropping Rice in South China

**DOI:** 10.3389/fpls.2017.00638

**Published:** 2017-04-28

**Authors:** Zhiyuan He, Xiaolei Qiu, Syed Tahir Ata-Ul-Karim, Yanda Li, Xiaojun Liu, Qiang Cao, Yan Zhu, Weixing Cao, Liang Tang

**Affiliations:** ^1^Jiangsu Key Laboratory for Information Agriculture, National Engineering and Technology Center for Information Agriculture, Jiangsu Collaborative Innovation Center for Modern Crop Production, Nanjing Agricultural UniversityNanjing, China; ^2^Institute of Agricultural Engineering, Jiangxi Academy of Agricultural SciencesNanchang, China

**Keywords:** late rice, early rice, critical nitrogen dilution curve, nitrogen nutrition index, shoot biomass, yield

## Abstract

The concept of critical nitrogen (*N*_c_) concentration can be implemented to diagnose in-season plant nitrogen (N) status for optimizing N fertilizer management. The *N*_c_ dilution curves have been established for rice (*Oryza sativa* L.) grown in different climatic regions, yet no attempt has been made to develop the *N*_c_ dilution curve for double cropping rice regions. This study was undertaken to develop the *N*_c_ dilution curves for double cropping rice in south China for assessment of in-season N status and to establish the relationships N nutrition index (NNI) and relative yield (RY) for in-season prediction of rice grain yield. Three different N application rate field experiments using six Indica rice varieties, including two early rice hybrids and four late rice hybrids were carried out in east China. The *N*_c_ dilution curves based on whole plant N concentration were determined and described as, N_c_ = 3.37 W^−0.44^ for early rice and N_c_ = 3.69 W^−0.34^ for late rice. The constant N concentration at early growth stage was 3.31 and 3.15% DM for early and late rice, respectively. Late rice showed a higher capacity of N accumulation and a lower rate of N decline per unit shoot biomass as compared to early rice. The curves for present study were different from the existing reference curves for Indica and Japonica rice grown in different rice growing regions. Integrated N nutrition index (NNI_int_) based on N_c_ was used to estimate RY at different growth periods using linear regression functions. The results showed that the critical curves and relationship between NNI_int_ and RY could be used as a reliable indicator of N status diagnosis, grain yield prediction as well as to provide technical support in N management for double cropping rice in south China.

## Introduction

Rice is one of the most important crops in China, and as the largest rice producer, China accounts for 28.1% of the global rice production (FAOSTAT., 2014). Approximately 34.6% of China's total rice production comes from the double cropping rice regions situated in south China (National Data, 2012)^1^. Excessive nitrogen (N) fertilizer application for optimizing crop production has resulted in a series of environmental problems such as soil acidification, eutrophication and greenhouse gas emissions (Ju et al., 2009). Moreover, excessive N application has also resulted in a low N use efficiency of 30–35% (Peng et al., 2006; Guo et al., 2010). Therefore, optimizing N fertilizer management to increase yields and reduce environmental problems has recently become a major research focus (Ata-Ul-Karim et al., 2014a; Yao et al., 2014).

Nitrogen status diagnosis during vegetative growth is a key technique for optimizing N fertilizer management (Ata-Ul-Karim et al., 2014b). There is an analytical method to diagnose N status based on the concept of critical nitrogen (N_c_), the minimum N concentration necessary to achieve maximum growth (Ulrich, 1952). The N_c_ represents the optimum N status in plants. If plant N concentration is higher than the N_c_ then N is in excess, if it is less, then N is insufficient. The N_c_ curve-based N nutrition index (NNI), the ratio of actual shoot N concentration (N_actual_) to N_c_, can also be used to diagnose the excess or deficiency of N in plants (Bélanger et al., 2001). It has been shown that plant N concentration within dense canopies decreases with increasing plant biomass, even when ample N is supplied. This phenomenon can be explained by plant aging and compartmentalization of metabolic and structural tissues (Lemaire and Gastal, 1997). Lemaire and Salette (1984) described this decline of N concentration as a negative power function based on the accumulated aerial biomass of lucerne:

(1) $$\begin{array}{r} {\text{N}}_{\text{c }}=\text{a}W^{-\text{b}} \end{array}$$

where *W* is aboveground biomass t ha^−1^, *N*_c_ is the N concentration in shoots expressed in % DM (dry matter), *a* is the N accumulation when *W* = 1 t ha^−1^ which is related to the amount of N supply and the intrinsic N absorption capacity of the crop during early growth stage. Parameter *b* is the decrease in the rate of N uptake with crop dry weight increase (Lemaire et al., 2007).

This concept has been established for over decades and N_c_ dilution curves based on shoot biomass have been developed in various crop species, including wheat (Justes et al., 1994; Ziadi et al., 2010; Yue et al., 2012) and rice (Sheehy et al., 1998; Ata-Ul-Karim et al., 2013; Huang et al., 2015). However, N_c_ dilution curves have not yet been developed or validated for double cropping rice areas of south China. The double cropping rice region dominated by Indica rice is one of the most important rice production regions in China, and contributes 34.6% of the national rice production. The region has a subtropical humid monsoon climate and produces several varieties using different management practices. Compared to single rice, the shorter growth period of early and late rice in the region results in faster crop growth rate, and early rice grows in a relative colder climatic condition (Huang et al., 2013). Moreover, the previous reports pointed out the interspecies and intraspecies dissimilarities in the N_c_ curve as well as between experimental sites (Justes et al., 1994; Bélanger et al., 2001), due to different morphological and histological characteristics (Lemaire and Gastal, 1997). Therefore, it is imperative to develop the appropriate N_c_ dilution curves for early and late rice for precise N diagnosis and yield optimization in the region.

This concept can potentially be implemented for guiding N dressing recommendation, predicting grain yield and N requirement (NR) in rice production, using quantitative relationships between relative yield (RY) and N_c_ dilution curve based N parameters (NNI, accumulated N deficit (AND) and NR) (Ata-Ul-Karim et al., 2016a, 2017a). Successful attempts have been also made for predicting grain yield in spring wheat, corn, sunflower (Ziadi et al., 2008, 2010; Debaeke et al., 2012). However, the relationships derived in previous studies were based on instantaneous NNI, either using the averaged NNI data at different crop growth stages or at particular crop growth stage, less attempts have been made to establish these relationships for RY and (Integrated NNI, NNI_int_) obtained by the weighted mean of NNI during the vegetative period. Previous reports indicated a linear relationship between NNI_int_ and relative biomass (actual dry weight divided by the maximum dry weight) (Lemaire and Gastal, 1997). Meanwhile, some researchers indicated that NNI_int_ can make a better estimation for grain yield during vegetative growth phase (Lemaire et al., 2008) and maize grain number per unit area is also highly correlated with NNI_int_ estimated during the period from seedling to 20 days after silking (Plénet and Cruz, 1997). We hypothesized that the relationship of RY with NNI_int_ for double cropping rice can better predict the grain yield being derived from actual in-season dry weight of rice crop.

Therefore, the present study was conducted to develop the N_c_ dilution curves based on shoot biomass for early and late rice in double cropping rice region of south China, to compare these curves with existing N_c_ dilution curves for Indica and Japonica rice and to determine the relationships for in-season estimation of rice grain yield. The projected results will provide technical support in precise diagnosing of in-season N status, fertilization guidance and yield forecasting for double cropping rice.

## Materials and methods

### Experimental design

Six different N fertilization treatments using 6 Indica rice varieties were conducted in Nanchang (28°33′N, 115°57′E), Jiangxi province of south China. Two early rice hybrids, including Zhongjiazao-17 (ZJZ-17) and Tanliangyou-83 (TLY-83) and four late rice hybrids, Tianyouhuazhan (TYHZ), Yueyou-9113 (YY-9113), Xiangyou-186 (XY-186), and Wufengyou-788 (WFY-788) were used. Detailed information about soil characteristics, cropping practices, climatic conditions, and N treatments in field experiments are summarized in Table 1. Experiments were arranged using a completely randomized block design with three replications. The banks between the individual plots were covered with plastic film to prevent fertilizer penetration across treatments. Two seedlings per hill were transplanted manually in all plots and the hill spacing was 0.24 m × 0.14 m and each plot area was 4 m × 5.4 m. The N fertilizer was applied as 60% before transplanting and 40% at tilling for early rice. For late rice it was 50% before transplanting, 30% at tilling and 20% at booting. Urea was used as the N fertilizer. Phosphorus (Ca(H_2_PO_4_)^2^) and potassium (KCl) fertilizers were added to the soil with the application rates of 60 kg ha^−1^ (P_2_O_5_) and 120 kg ha^−1^ (K_2_O) before transplanting.

**Table 1:** **Basic information about the field experiments conducted during the study period**.

**Experiment no. and locations**	**Rice type and variety**	**Transplanting and harvest date**	**N rate (kg•ha^−1^)**	**Sampling date**	**Growth stage**	**Soil characteristics**	**Average temperature (transplanting to flowering)**
Exp. 1 2013 Nanchang 28°33'N, 115°57'E	Early rice ZJZ-17 TLY-83	26-April 11-July	N0 (0) N1 (75) N2 (150) N3 (225)	14-May 21-May 04-June 09-June 24-June	MT SE PI BT HD	*OM* = 16.6 g kg^−1^ Total *N* = 1.3 g kg^−1^ Available *P* = 9.1 mg kg^−1^ Available *K* = 77 mg kg^−1^	24.58°C
	Late rice TYHZ YY-9113	29-July 28-Oct.	N0 (0) N1 (90) N2 (180) N3 (270)	19-Aug 28-Aug 02-Sep. 09-Sep. 16-Sep.	MT SE PI BT HD	*OM* = 18.5 g kg-1 Total *N* = 1.3 g kg-1 Available *P* = 8.4 mg kg^−1^ Available *K* = 56 mg kg-1	30.48°C
Exp. 2 2014 Nanchang 28°33'N, 115°57'E	Early rice ZJZ-17 TLY-83	24-April 13-July	N0 (0) N1 (75) N2 (150) N3 (225) N4 (300)	15-May 21-May 27-May 03-June 11-June 16-June	AT MT SE PI BT HD	*OM* = 20.6 g kg^−1^ Total *N* = 1.9 g kg^−1^ Available *P* = 7.9 mg kg^−1^ Available *K* = 42 mg kg^−1^	24.21°C
	Late rice TYHZ YY-9113	27-July 22-Oct.	N0 (0) N1 (90) N2 (180) N3 (270) N4 (360)	12-Aug 20-Aug 27-Aug 04-Sep. 11-Sep. 23-Sep.	AT MT SE PI BT HD	*OM* = 20.7 g kg^−1^ Total *N* = 1.7 g kg^−1^ Available *P* = 6.9 mg kg^−1^ Available *K* = 34 mg kg^−1^	28.87°C
Exp. 3 2015 Nanchang 28°33'N, 115°57'E	Early rice ZJZ-17 TLY-83	30-April 13-July	N0 (0) N1 (75) N2 (150) N3 (225) N4 (300)	17-May 22-May 27-May 05-June 11-June 16-June	AT MT SE PI BT HD	*OM* = 21.7 g kg^−1^ Total *N* = 1.4 g kg^−1^ Available *P* = 7.12 mg kg^−1^ Available *K* = 46 mg kg^−1^	23.99°C
	Late rice XY-186 WFY-788	20-July 26-Oct.	N0 (0) N1 (90) N2 (180) N3 (270) N4 (360)	12-Aug 24-Aug 27-Aug 04-Sep. 11-Sep. 16-Sep.	AT MT SE PI BT HD	*OM* = 28.8 g kg^−1^ Total *N* = 1.5 g kg^−1^ Available *P* = 6.24 mg kg^−1^ Available *K* = 65 mg kg^−1^	29.52°C

### Sample collection and analysis

Five hills were sampled from each plot at active tillering (AT), mid tillering (MT), stem elongation (SE), panicle initiation (PI), booting (BT), and heading (HD) stages during the vegetative phase for growth analysis. The sampling dates are detailed in Table 1. Fresh samples were separated into leaves and stems. All samples were oven-dried at 105°C for 30 min to stop metabolism and then at 80°C until constant weight was reached to determine shoot biomass (t ha^−1^). All samples were milled and analyzed for total shoot N concentration by the micro-Kjeldahl method.

### Data analysis

#### Establishment and validation of critical N dilution curve

Data from Experiments 1 and 2 (Table 1) were used to develop the N_c_ dilution curve following the computation method of Justes et al. (1994). The data points for which N did not limit growth (non-N-limiting growth) or was not in excess (N-limiting growth) were identified from Experiments 1 and 2. In order to calculate the critical values, defined as the intersection of a vertical line and an oblique line, the differences between treatment means were assessed using least significant difference (LSD 0.10) test, instead of classically using 0.1 in order to reduce the occurrence of Type II errors (i.e., the error of incorrectly retaining a false null hypothesis) (Ata-Ul-Karim et al., 2016b). The results were used to classify N-limiting and non-N-limiting growth treatments. A non-N-limiting growth treatment was defined as a treatment for which N application did not lead to an increase in shoot biomass but did significantly increase shoot N concentration. If at the same measurement date, statistical analysis distinguished at least one set of N limiting growth and non-N-limiting growth data points, these data were used to define the N dilution curve. The constant N_c_ concentration at early growth stages in early and late rice was determined by calculating the mean value between the minimum N concentration of non-limiting N points and the maximum N concentration of limiting N points. Data collected from the independent experiments (Exp. 3) were used to validate the *N*_c_ dilution curves.

#### Statistical analysis

For each sampling date, experiment, rice type, the amount of shoot biomass produced with the varied N treatments and the corresponding N concentrations were subjected to analysis of variance (ANOVA) using GLM procedures in SPSS-16 (SPSS Inc., Chicago. IL, USA). Analysis of covariance (ANCOVA) at the 90% confidence interval was used to define the significance of N_c_ dilution curves of early rice and late rice. Differences were considered significant at *Sig* < 0.1 at the 90% confidence interval. The coefficient of determination (R^2^), relative root mean-squared error (RRMSE) and accuracy (the slope of the linear regression equation between the estimated and intercepted values at zero intercept) were used to evaluate the model estimated effect.

#### Determination of NNI, integrated NNI, and relative yield

The NNI at each sampling date was calculated according to Justes et al. (1994) using Equation (2).

(2) $$\begin{array}{r} \text{NNI }={\text{N}}_{\text{actual}}/{\text{N}}_{\text{c}} \end{array}$$

Where N_actual_ and N_c_ are the actual N concentration and critical N concentration in the shoot, respectively. If NNI = 1, it represents optimum N nutrition, while NNI > 1 and NNI < 1 indicates excess and deficient N nutrition, respectively.

An integrated NNI can be obtained by the weighted mean of NNI during the vegetative period (Lemaire et al., 2008) using Equation (3):

(3) $$\begin{array}{r} \text{NN}{\text{I}}_{\text{int}}=1/T\sum \text{NN}{\text{I}}_{\text{i}}\times t_{\text{i}} \end{array}$$

Where NNI_int_ is the integrated NNI, *T* is time (days or GDD), NNI_i_ is instantaneous NNI values for different sampling periods and *t*_i_ is the interval time.

The relative yield (RY) for each experimental site was calculated using Equation (4).

(4) $$\begin{array}{r} \text{Relative yield}=\text{G}{\text{Y}}_{\text{treatment}}/\text{G}{\text{Y}}_{\text{max}}\times 100 \end{array}$$

Where GY_treatment_ is the actual yield of each N treatment; GY_max_ is the mean of the yield for the group of treatments giving the highest yield value (LSD < 0.1).

The regressions were used to estimate relationships between shoot biomass and N concentration and between NNI_int_ and RY using IBM SPSS version 16.0 software.

## Results

### Shoot biomass and N concentration

The production of shoot biomass of both early and late rice varieties was significantly affected by N application. The increase in shoot biomass followed a gradually increasing trend after transplanting for early and late rice varieties in each experiment with increasing N application rates. However, non-significant differences were observed between N application rates of 225 and 300 kg ha^−1^ for early rice and late rice. The shoot biomass for early rice ranged from 0.21 to 10.86 t ha^−1^ (ZJZ-17) and 0.23 to 10.50 t ha^−1^ (TLY-83) in 2013, and 0.34 to 10.16 t ha^−1^ (ZJZ-17) and 0.34 to 8.78 t ha^−1^ (TLY-83) in 2014. For late rice, the shoot biomass of TYHZ ranged from 1.64 to 14.84 t ha^−1^ in 2013 and 0.83 to 14.5 t ha^−1^ in 2014. For YY-9113 shoot biomass ranged from 1.13 to 13.9 t ha^−1^ in 2013 and 1.02 to 14.2 t ha^−1^ in 2014 (Table 2). Overall, the shoot biomass of early rice varieties (ZJZ-17, TLY-83) was slightly lower than late rice varieties (TYHZ, YY-9113).

**Table 2:** **Shoot biomass on different sampling dates under different N treatments**.

**Early rice**	**Sampling date**	**Applied N Kg ha**^**−1**^					**F prob**.	**LSD**
		**0**	**75**	**150**	**225**	**300**		
ZJZ-17 2013	14-May	0.21b	0.28a	0.30a	0.32a		^*^	0.05
	21-May	0.57c	0.69b	0.72b	1.02a		^*^	0.21
	04-June	2.44d	3.98c	3.33b	4.32a		^*^	0.76
	09-June	4.34b	5.40a	5.96a	5.55a		^*^	1.25
	24-June	7.67c	10.02b	9.97b	10.86a		^*^	1.56
TLY-83 2013	14-May	0.23a	0.26a	0.30a	0.43b		^*^	0.05
	21-May	0.68c	0.74b	0.72b	0.80c		^*^	0.10
	04-June	2.63d	2.90c	3.78b	4.23a		^*^	1.11
	09-June	4.83d	5.05c	5.21b	6.12a		^*^	2.74
	24-June	8.12d	9.27c	10.50b	11.97a		^*^	1.89
ZJZ-17 2014	15-May	0.34c	0.62b	0.72a	0.75a	0.76a	^*^	0.07
	21-May	0.55c	1.03b	1.20a	1.24a	1.30a	^*^	0.13
	27-May	1.34c	2.22b	2.96a	3.11a	3.05a	^*^	0.35
	03-June	2.21c	3.70b	4.43a	4.69a	4.96a	^*^	0.43
	11-June	3.52c	5.17b	6.53a	6.78a	7.13a	^*^	1.01
	16-June	4.14d	6.86c	8.47b	9.28ab	10.16a	^*^	0.92
TLY-83 2014	15-May	0.34b	0.59a	0.74a	0.72a	0.59a	^*^	0.16
	21-May	0.55b	0.90a	1.01a	0.97a	1.00a	^*^	0.15
	27-May	1.21c	1.87b	2.56a	2.57a	2.82a	^*^	0.39
	03-June	2.17c	3.42b	4.08b	4.87a	5.29a	^*^	0.60
	11-June	3.33c	4.79b	5.97a	6.86a	6.71a	^*^	0.81
	16-June	3.40d	5.37c	7.18b	8.19a	8.78a	^*^	0.60
**Late rice**	**Sampling dates**	**Applied N Kg ha**^−1^					**F prob**.	**LSD**
		**0**	**90**	**180**	**270**	**360**		
TYHZ 2013	19-Aug.	1.64c	2.21b	2.81a	2.69a		^*^	0.71
	28-Aug.	2.77d	3.38c	4.42b	6.42a		^*^	0.68
	02-Sep.	4.23d	5.82c	6.30b	8.42a		^*^	0.63
	09-Sep.	5.39d	7.64c	8.94b	10.98a		^*^	1.08
	16-Sep.	8.49d	9.32c	11.45b	13.45a		^*^	1.68
	24-Sep.	9.93c	11.96b	13.59a	14.84a		^*^	1.78
YY-9113 2013	19-Aug.	1.13d	1.79c	1.82b	2.14a		^*^	0.70
	28-Aug.	2.18d	3.13c	3.45b	4.53a		^*^	0.72
	02-Sep.	3.41c	4.71b	6.23a	6.69a		^*^	0.99
	09-Sep.	4.81c	7.10b	7.42b	8.20a		^*^	1.16
	16-Sep.	6.01d	9.44c	11.51b	12.40a		^*^	1.73
	24-Sep.	7.46d	11.13c	12.52b	13.90a		^*^	1.03
TYHZ 2014	12-Aug.	0.83c	0.98c	1.20b	1.32ab	1.47a	^*^	0.18
	20-Aug.	1.47b	2.03a	2.21a	2.19a	2.19a	^*^	0.25
	27-Aug.	2.99b	4.17a	4.31a	4.57a	4.56a	^*^	0.66
	04-Sep.	5.74d	7.31c	8.62b	9.28a	9.57a	^*^	1.27
	11-Sep.	7.44d	9.29c	9.90b	10.73a	11.01a	^*^	0.44
	16-Sep.	7.81c	10.21b	10.93b	11.85a	12.30a	^*^	1.26
	23-Sep.	8.78c	11.29b	12.62b	14.47a	14.50a	^*^	1.28
YY-9113 2014	12-Aug.	1.02c	1.20b	1.28a	1.32a	1.31a	^*^	0.20
	20-Aug.	1.45d	1.84c	2.19b	2.51ab	3.01a	^*^	0.53
	27-Aug.	2.73c	3.72b	3.87ab	4.20ab	4.37a	^*^	0.48
	04-Sep.	4.78c	6.50b	7.68a	7.90a	8.29a	^*^	0.79
	11-Sep.	7.60c	8.99b	10.08a	10.59a	11.92a	^*^	2.19
	16-Sep.	8.69d	9.70c	10.96b	12.52a	12.22a	^*^	1.97
	23-Sep.	10.47c	12.10b	12.72b	14.20a	13.70a	^*^	1.72

* *F statistic significant at the 0.10 probability level*.

*LSD, least significant difference*.

*Different letter exhibit the significance differences between the treatments at the 0.10 probability level*.

**Figure 1:**
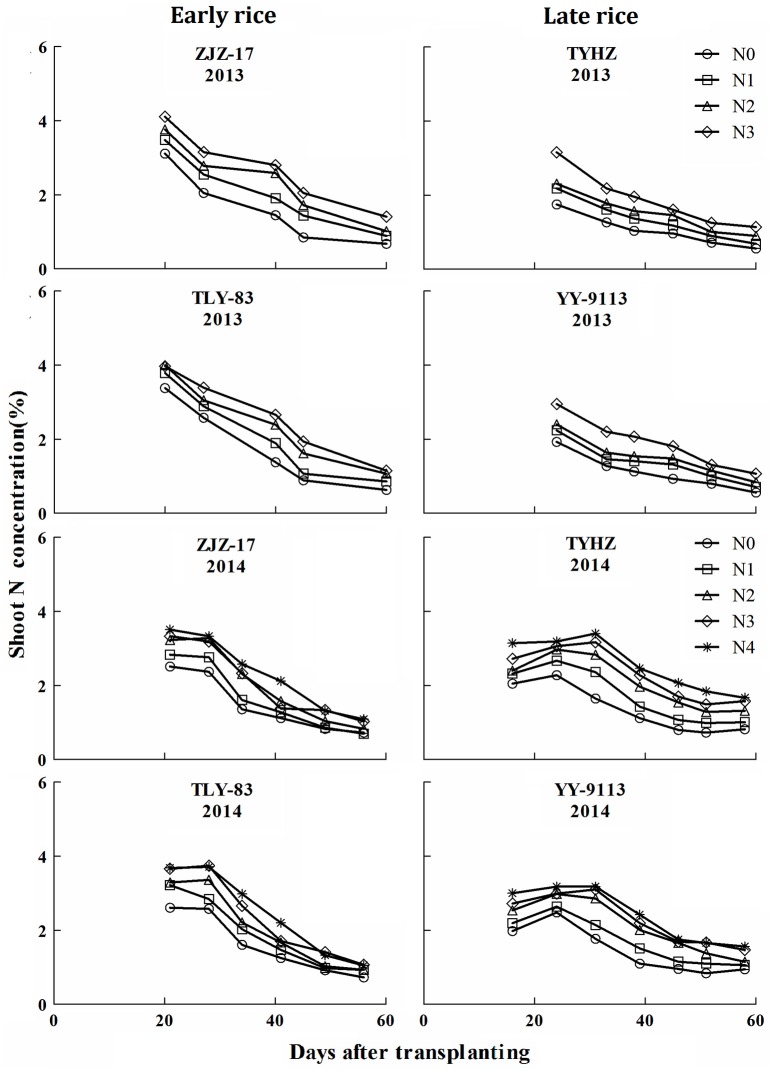
**N concentration (% DM) under varied N application rates with days after transplanting in experiments conducted during 2013 and 2014 in early and late rice varieties**.

The response of plant N concentration to N application rates was generally linear and a higher rate of N application generally resulted in a higher plant N concentrations. A decline in plant N concentrations was observed with increasing shoot biomass. The Figure 1 showed that the plant N concentrations in early rice varieties ranged from 0.85 to 4.11 (ZJZ-17) and 0.85 to 4.00% (TLY-83) in 2013 and 0.70 to 3.51 (ZJZ-17) and 0.73 to 3.68% (TLY-83) in 2014. For late rice varieties, the N concentration ranged from 0.56 to 3.16 (TYHZ) and 0.56 to 2.95% (YY-9113) in 2013 and 0.82 to 3.40 (TYHZ) and 0.84 to 3.18% (YY-9113) in 2014. The early rice varieties showed higher plant N concentrations than the late rice varieties in the early growth stages and similar trends were observed in later stages.

### Determination of N_c_ dilution curve

**Figure 2:**
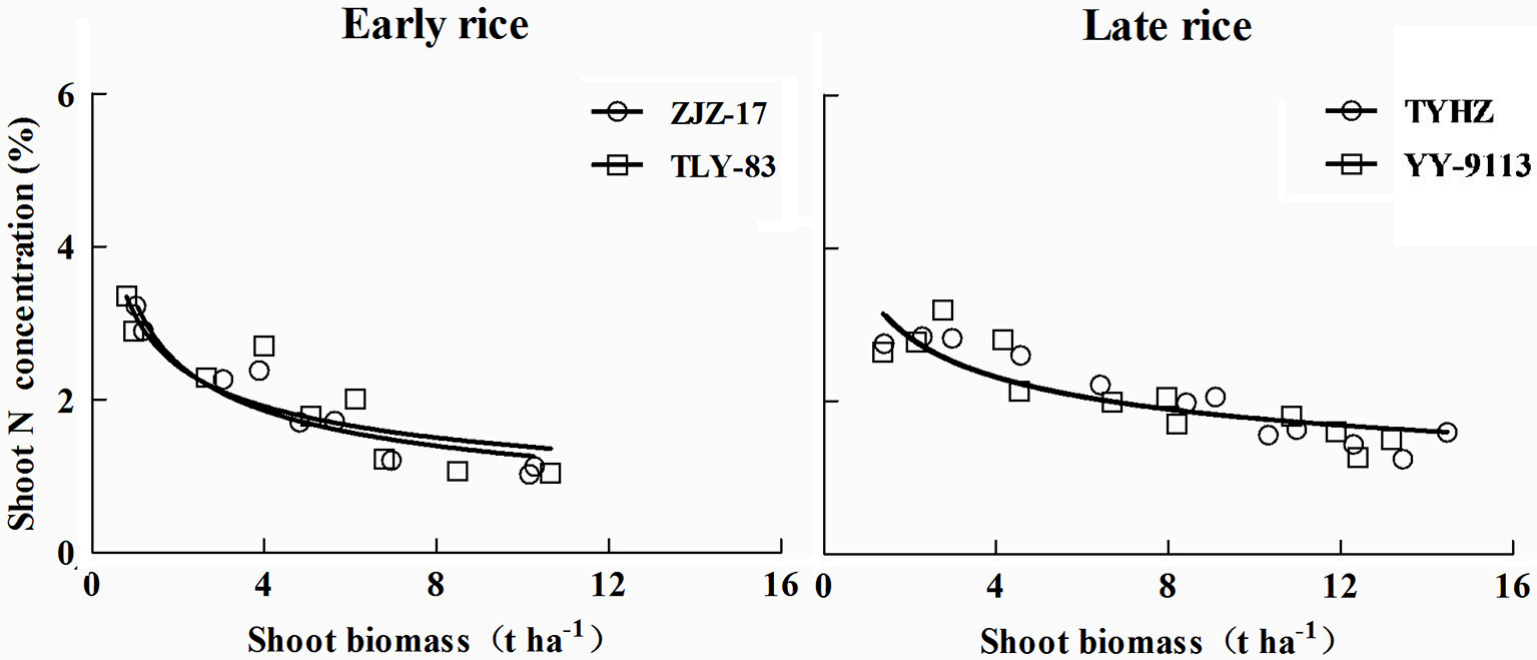
**Critical dilution curves of early rice (ZJZ-17, N_**c**_ = 3.49 W^**−0.47**^, ***R***^**2**^ = 0.8982 and TLY-83, N_**c**_ = 3.28 W^**−0.42**^, ***R***^**2**^ = 0.7661) and late rice varieties (TYHZ, N_**c**_ = 3.74 W^**−0.34**^, ***R***^**2**^ = 0.8025 and YY-9113, N_**c**_ = 3.66 W^**−0.33**^, ***R***^**2**^ = 0.7632) in experiment conducted during 2013 and 2014**.

A total of 18 data points of early rice and 24 data points of late rice obtained from experiment 1 and 2 were used to define the N_c_ dilution curves. The shoot biomass data for developing the N_c_ dilution curves ranged from 0.8 to 10.65 t ha^−1^ for early rice and 1.37–14.48 t ha^−1^ for late rice. The N_c_ dilution curves of early and late rice varieties established during present study were shown in Figure 2.

Analysis of covariance (ANCOVA) at the 90% confidence interval was used to define the significance of N_c_ dilution curves of early rice and late rice. The values *Sig*. of slope and intercept in Table 3 for early rice (ZJZ-17 and TLY-83) are greater than 0.1 showed that there is no statistical difference between the two varieties for early rice and late rice.

**Table 3:** **Validation NNI_**int**_ and RY models at different stages for early and late rice**.

**Sig**.	**ZJZ-17 TLY-83**	**TYHZ YY-9113**	**Early rice Late rice**
Slope	0.551	0.720	0.000
Intercept	0.974	0.843	0.000

**Figure 3:**
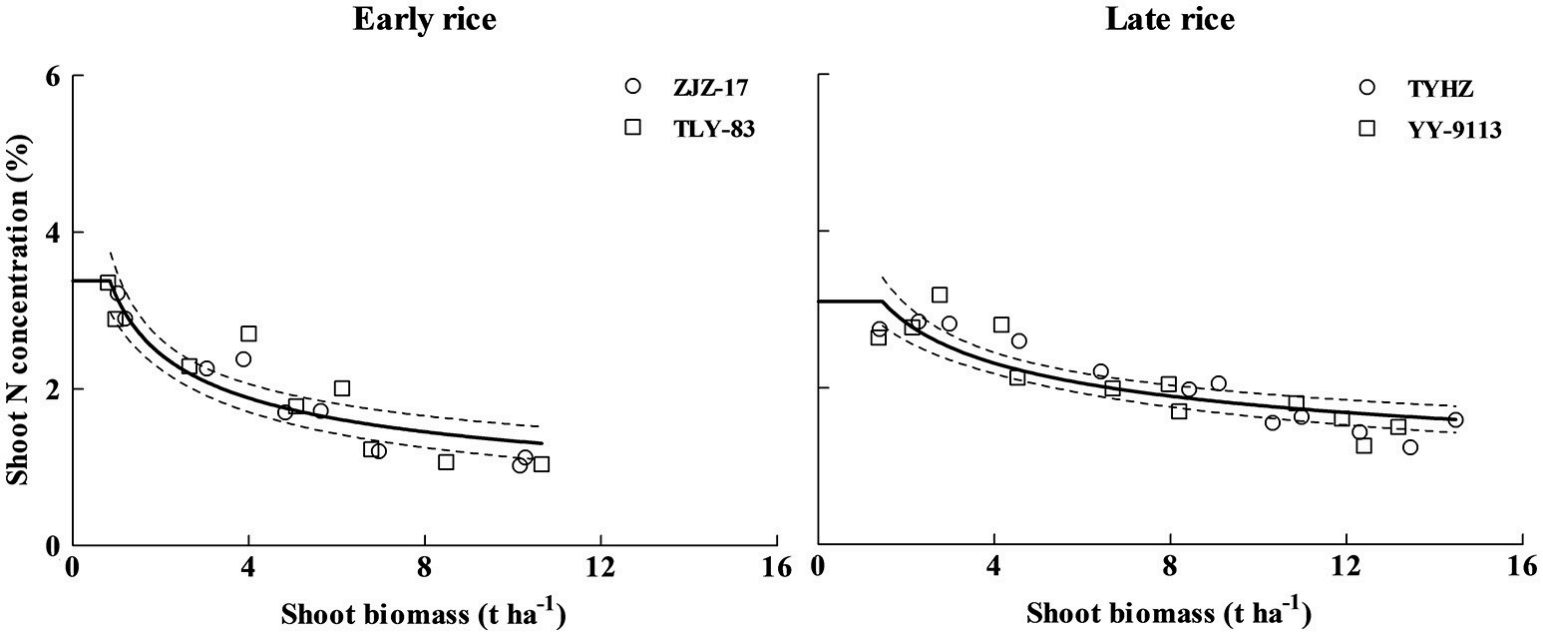
**Unified N_**c**_ dilution curves of early rice (N_**c**_ = 3.37 W^**−0.44**^***R***^**2**^ = 0.8259) and late rice (N_**c**_ = 3.69 W^**−0.34**^***R***^**2**^ = 0.7834) in experiments conducted during 2013 and 2014**. The solid line denotes the power function regression and the dotted lines represent the confidence bands (*p* = 0.95). The solid lines parallel to x-axis represent the constant N concentration during early growth stage, the intersection between power function curve and linear line are (1.04 t ha^−1^, 3.31%DM) and (1.6 t ha^−1^, 3.15%DM) for early and late rice respectively.

The results indicated that growth rate and varieties don't significantly affect N_c_ for each rice type. However, significant differences were observed between early and late rice. The data of different varieties for each rice type were pooled, and the unified dilution curves for early and late rice were determined (Figure 3).

(5) $$\begin{array}{r} {\text{N}}_{\text{cEarly}}=3.37W^{-0.44}\;\left(\text{Early rice};\text{W}\geq 0.8\text{t h}{\text{a}}^{-1},{\text{R}}^{2}=0.8259\right) \end{array}$$

(6) $$\begin{array}{r} {\text{N}}_{\text{cLate}}=3.69W^{-0.34}\;\left(\text{Late rice};\text{W}\geq 1.37\text{t h}{\text{a}}^{-1},{\text{R}}^{2}=0.7834\right) \end{array}$$

Where N_cEarly_ and N_cLate_ are the N_c_ concentration in shoot biomass expressed in % DM for early and late rice, respectively, *W* is the shoot biomass (t ha^−1^). Parameter *a* for early rice (3.37) was lower than late rice (3.69). In contrast, parameter *b* of early rice (0.44) was higher than that of late rice (0.34).

The 95% confidence interval of the plant N concentration was 2.92–4.74 and 2.92–3.78% DM for a shoot biomass of 0.8 and 1.37 t ha^−1^, for early and late rice, respectively. For shoot biomass 9.72 (early rice) and 14.79 (late rice) t ha^−1^, the 95% confidence interval of the N_c_ concentration in aerial tissues was 1.06–1.32 and 1.37–1.64% DM for early and late rice, respectively (Figure 3). Eight data points ranging from 0.51 to 1.2 t ha^−1^ and 12 data points ranging from 0.26 to 0.80 t ha^−1^ for early and late rice were used to determine the constant N concentration for early and late rice respectively. The constant N_c_ concentrations, 3.31%DM (1.04 t ha^−1^) and 3.15%DM (1.6 t ha^−1^), respectively for early and late rice were calculated as the mean value between the minimum N concentration of N non-limiting points and the maximum N concentration of N limiting points.

### Validation of the N_c_ dilution curve

**Figure 4:**
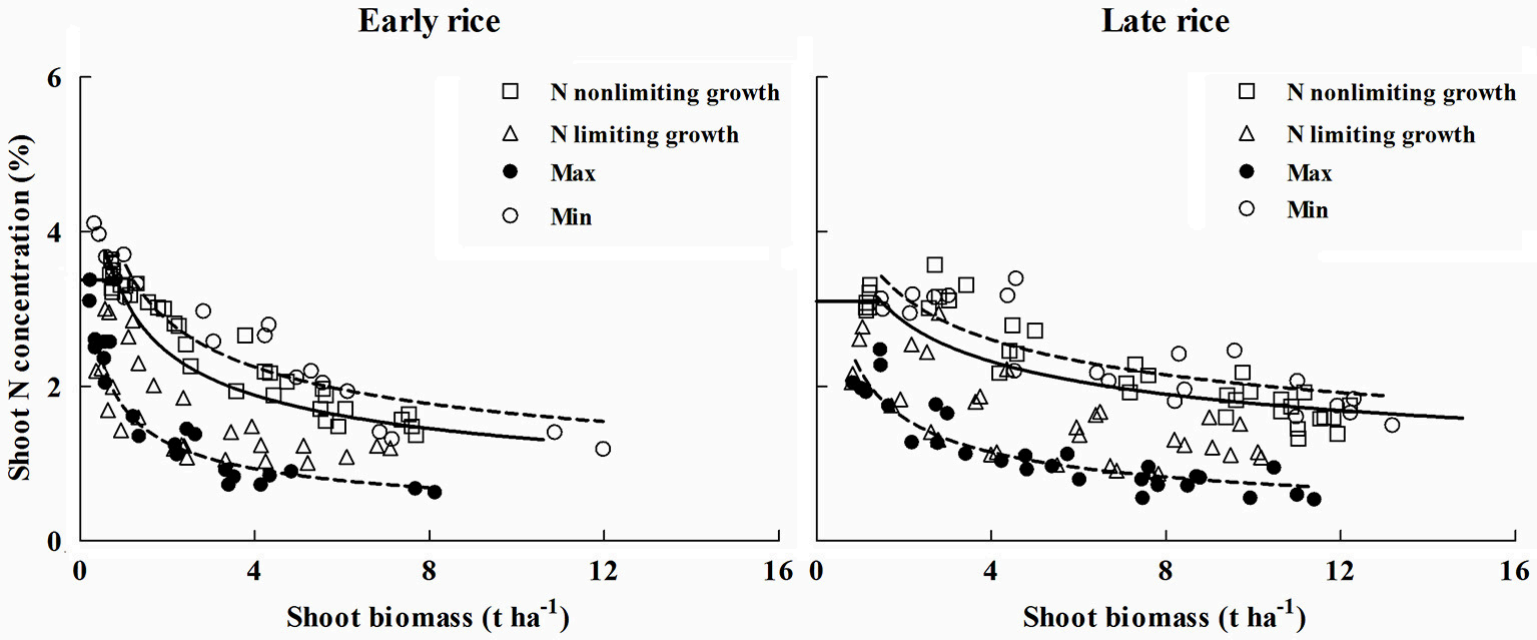
**Validation of the N_**c**_ dilution curves for early rice and late rice**. Data points (□) represent non-N-limiting condition, (Δ) represent N limiting condition. The solid line indicates the N_c_ curves (N_c_ = 3.37 W^−0.44^; N_c_ = 3.69 W^−0.34^) for early and late rice, respectively, while the dashed lines represent the minimum and maximum N curves, (early rice: N_min_ = 1.72 W^−0.48^, N_max_ = 4.01 W^−0.43^; late rice: N_min_ = 2.29 W^−0.52^, N_max_ = 4.01 W^−0.31^). The (°) points represent N concentration from highest N rate and the (•) points are from zero N rate obtained from Experiments 2–3.

The N_c_ dilution curves of early and late rice were validated with a dataset obtained from independent experiments (Exp. 3). The data points under different N treatments from the independent experiments were categorized into N limiting or non-N-limiting growth conditions based on significant (*P* ≤ 0.1) differences in shoot biomass for each sampling date, site, and year. Treatments were considered N-limiting when shoot biomass significantly increased with increasing N supply, while non-N-limiting treatments had no significant increase in biomass with increasing N supply (LSD < 0.1). Data points acquired from N limiting treatments were positioned approximately below the critical curves while those of non-N-limiting treatments were positioned close to or above the critical curves (Figure 4). The N_c_ dilution curves of early and late rice differentiate well between the N limiting and non-N-limiting conditions.

Due to an obvious variation in plant N concentration for a given shoot biomass, N maximum (N_max_) and minimum (N_min_) curves were determined for both rice types.

(7) $$\begin{array}{r} \text{Early rice}:{\text{N}}_{\text{max}}=4.01W^{\;-0.43} \end{array}$$

(8) $$\begin{array}{r} {\text{N}}_{\text{min}}=1.72W^{\;-0.48} \end{array}$$

(9) $$\begin{array}{r} \text{Late rice}:{\text{N}}_{\text{max}}=4.01W^{\;-0.31} \end{array}$$

(10) $$\begin{array}{r} {\text{N}}_{\text{min}}=2.29W^{\;-0.52} \end{array}$$

The data points from the highest N treatments represent the maximum N dilution curve (N_max_), and the zero N application represents the minimum N dilution curve (N_min_) (Figure 4).

Plant N concentration varies between 1.96% below and 1.19% above the N_c_ dilution curve for early rice, 1.61% below and 1.09% above the N_c_ dilution curve for late rice.

### Estimation of nitrogen nutrition index

**Figure 5:**
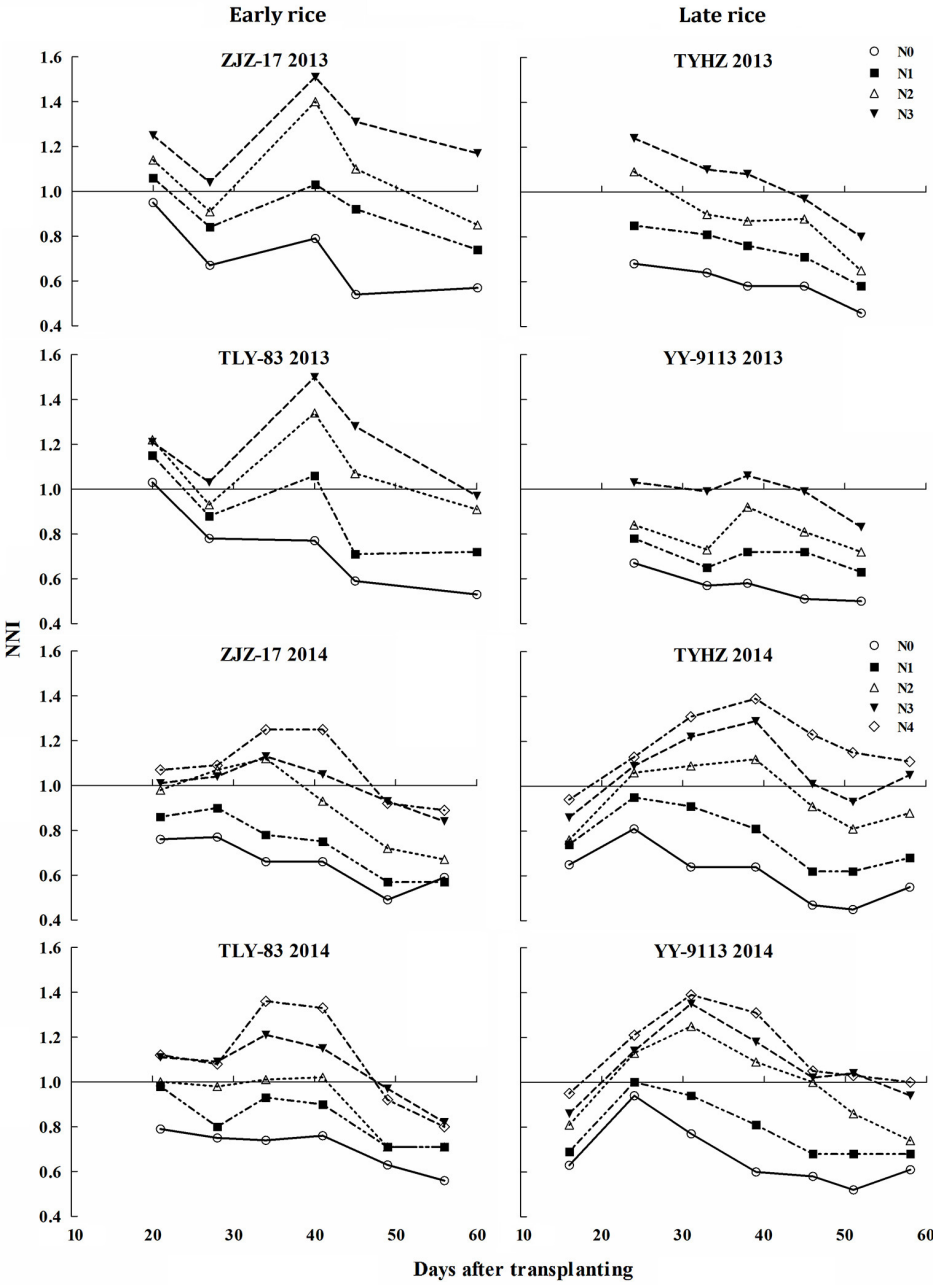
**Changes of nitrogen nutrition index (NNI) under various nitrogen (N) application rates in experiments conducted during 2013 and 2014**.

The results showed significant differences across the N treatments, growing seasons, crop growth stages, and early and late rice. The NNI values ranged from 0.49 to 1.51 and 0.45 to 1.53 for early and late rice, respectively (Figure 5). The NNI values of early and late rice varieties increased under different N_c_ treatments till 35 days after transplanting and then gradually decreased. The NNI values for early rice under N0 were generally below 1. For N2 to N4 values were generally above 1 and fluctuated near 1 for N1. In contrast, the NNI values for late rice showed differences between the two experiments. NNIs of N3 were closed to 1 in Exp. 1, but in Exp. 2, N2 was the optimal treatment.

### Establishment and validation of the relationship between integrated NNI and RY

**Figure 6:**
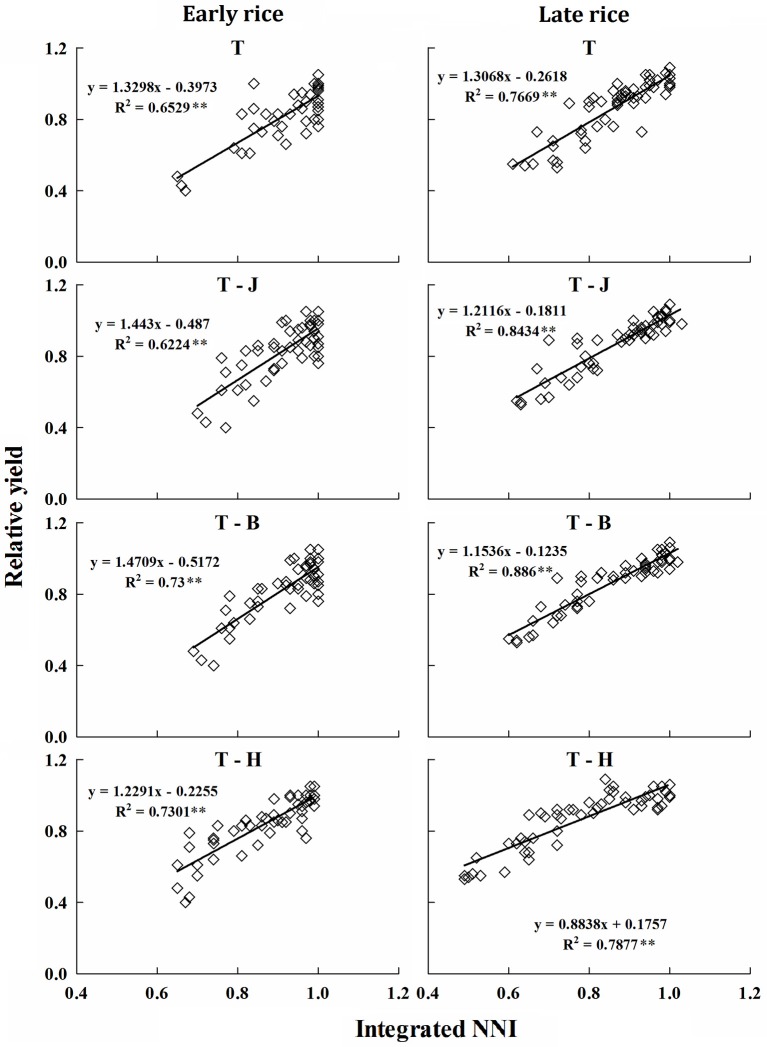
**Relationship between NNI_***int***_ and RY at different growth periods for early and late rice in experiments conducted during 2013 and 2014**. The T, J, B, and H represent growth stage of tillering, jointing, booting and harvest respectively. ^**^Significant at *p* < 0.01.

The RY was expressed as a function of NNI_int_ at different growth periods tillering (T), tillering to jointing (T-J), tillering to booting (T-B), and tillering to harvest (T-H) of early and late rice, and the relationships between NNI_int_ and RY of different periods indicated a close linkage between two parameters for early and late rice. The results showed that NNI_int_ and RY had a significant linear relationship and RY increased with increasing NNI_int_ (Figure 6). R^2^ of early and late rice models were between 0.62–0.73 and 0.77–0.89. Slight differences were observed between the slopes and intercepts of the linear functions at different stages and between the early and late rice models.

The robust relationships at different crop growth periods accurately explained the variation in RY both under N-limiting and non-N-limiting growth conditions. Therefore, the present relationships between RY and NNI_*int*_ are of practical meaning for in-season estimation of grain yield in double cropping rice varieties.

The results in Table 4 show that models at each growth period offer a good prediction of yield in the independent experiments. The R^2^, RRMSE and accuracy ranged from 0.8451 to 0.9004, 6.896 to 8.811, and 0.8174 to 1.0163%, respectively, for early rice. For late rice R^2^, RRMSE and accuracy ranged from 0.818 to 0.9332, 4.848 to 9.421%, and 1.0131 to 1.251, respectively.

**Table 4:** **Validation NNI_**int**_ and RY models at different stages for early and late rice**.

**Rice type**	**Growth stage/period**	**Data(n)**	***R*^2^**	**RRMSE (%)**	**Accuracy**
Early rice	T	30	0.8732	8.811	0.9152
	T-J	30	0.8945	8.127	1.0158
	T-B	30	0.9004	8.134	1.0163
	T-H	30	0.8451	6.896	0.8174
Late rice	T	30	0.818	8.168	1.1246
	T-J	30	0.9043	9.421	1.251
	T-B	30	0.9244	5.507	1.188
	T-H	30	0.9332	4.848	1.0131

## Discussion

### Differences of N concentration in aerial biomass for early rice and late rice

In this study, N concentration decreased with increasing shoot biomass. Results of N concentration and shoot biomass were in consensus with previous reports (Lemaire and Gastal, 1997; Ziadi et al., 2010; Ata-Ul-Karim et al., 2013). This phenomenon of decreasing N is mainly attributed to plant aging and phenology (Lemaire et al., 2007). Sheehy et al. (1998) suggested that the internal cycling of N from old to young developing tissues and an aging root system may play an important role in the rate of N accumulation and N concentration. However, in this study a sudden change in plant N concentration was observed at the jointing stage in 2014 for late rice (Figure 1). This is likely because sampling was conducted 1 week after N addition resulting in increases in N concentration. This is similar to the report of Justes et al. (1994) that N supplying resulted in an increase in the N concentration. The constant N concentration for both early and late rice was likely the result of shoot biomass increasing and the lack of competition for light among isolated plants in initial growth stage as reported by Lemaire and Gastal (1997).

There was a larger variability of N concentration for early rice than that of late rice. However, the lower shoot biomass in early rice was attributed to lower accumulated temperatures and less sunshine in the early rice season (Huang et al., 2013). The difference in N concentration in biomass can be explained by the specific value for the two rice types. The proportionality coefficient *k* linking N uptake and growth rate using mathematical method (Justes et al., 1994) as follows:

(11) $$\begin{array}{r} \frac{dN_{a}}{dt}=k\left(\frac{dW}{dt}\right) \end{array}$$

where *N*_a_ represents the amount of N uptake in the shoot expressed in kg ha^−1^ and *W* is the shoot biomass expressed in t ha^−1^. The *k* values for early rice (*k*_*early*_) and late rice (*k*_*late*_) calculated according to the Equations (4) and (5) were, *k*_*early*_ = 18.9 W^−0.44^ and *k*_*late*_ = 24.4 W^−0.34^, respectively. The results showed that *k*_*early*_ < *k*_*late*_, validated from 0.8 to 10.65 t ha^−1^ for early rice and from 1.37 to 14.48 t ha^−1^ for late rice. Coefficient k depends on the shoot biomass accumulation and the N absorption rate is directly related to the growth rate and biomass (Justes et al., 1994), k_early_ < k_late_ was validated in whole vegetable growth stage, thus the biomass accumulation of early rice is lower than late rice, and we can infer that N absorption rate of early rice is lower than late rice. Thus, the results from the coefficient *k* imply that early rice has a lower N accumulation capacity in shoots than late rice for the same aerial biomass.

The variability of the maximum and minimum N dilution curves from the critical curve was 0.77 and 0.52 for early and late rice, respectively. These values are very similar to those for Japonica rice 0.77 (1.8% below and 1.03% above) (Ata-Ul-Karim et al., 2013). Similar variability in plant N concentration for Indica and Japonica rice show that both rice ecotypes have similar capacity for N absorption from minimum to maximum N concentrations in their shoots. The N_max_ curve represents the maximum capacity of N accumulation in the shoot, while the N_min_ curve represents the lower limit at which metabolism would cease to function (Justes et al., 1994).

### Comparison of critical nitrogen dilution curves

Critical N dilution curves have been previously developed for Japonica and Indica rice ecotypes under different climatic conditions (Sheehy et al., 1998; Ata-Ul-Karim et al., 2013; Huang et al., 2015). The existing N_c_ dilution curves were compared with the newly developed curves of early and late rice in the present study (Figure 5). The parameter information of these curves is presented in Table 5.

**Table 5:** **The location, variety and parameter of critical N dilution curve for rice grown in different environments**.

**Type**	**Variety**	**Location**	**Transplanting date**	**Climate zone**	**Critical curve N = *aW^−*b*^***		
					***a***	***b***	**References**
Indica rice	Early rice (ZJZ-17, TLY-83)	Jiangxi (28°33′N, 115°57′E,)	27-April	Subtropical humid monsoon	3.37	0.44	This study
	Late rice (TYHZ, YY-9113)	Jiangxi (28°33′N, 115°57′E,)	29-July		3.69	0.34	This study
	IR72	Philippines (IRRI),		Tropical	5.20	0.50	Sheehy et al., 1998
Japonica rice	WXJ-14, LXY-18	Yizheng (32°16′N, 119°10′E,)	20-June	Subtropical-temperate	3.53	0.28	Ata-Ul-Karim et al., 2013
	Kendao, Longjing	Heilongjian (47°14′N, 132°49′E,)	17-May	Cool-temperate sub-humid continental monsoon climate	2.77	0.34	Huang et al., 2015

The coefficients of N_c_ dilution curves for Indica rice showed significant differences between the three Indica rice types. This indicates that Indica rice has different rates of N uptake per unit biomass accumulated, which may be due to temperature difference, day length and crop growth rate. In the early rice growing season (transplanting to flowering), the average temperature was about 6°C lower than that in the late rice growing season. Previous studies have shown that temperature has a significant effect on the absorption of N in rice. Low temperatures result in reduced N uptake and relatively higher temperatures lead to higher N uptake (Shimono et al., 2012), which may explain the lower parameter *a* and higher parameter *b* for early rice. The peculiarity of IR72 is that the N concentration at early growth stage is higher than that of late rice but at late growth period the N concentration is close to late rice, then faster N declined with increasing biomass and leading to a higher parameter *b*.

**Figure 7:**
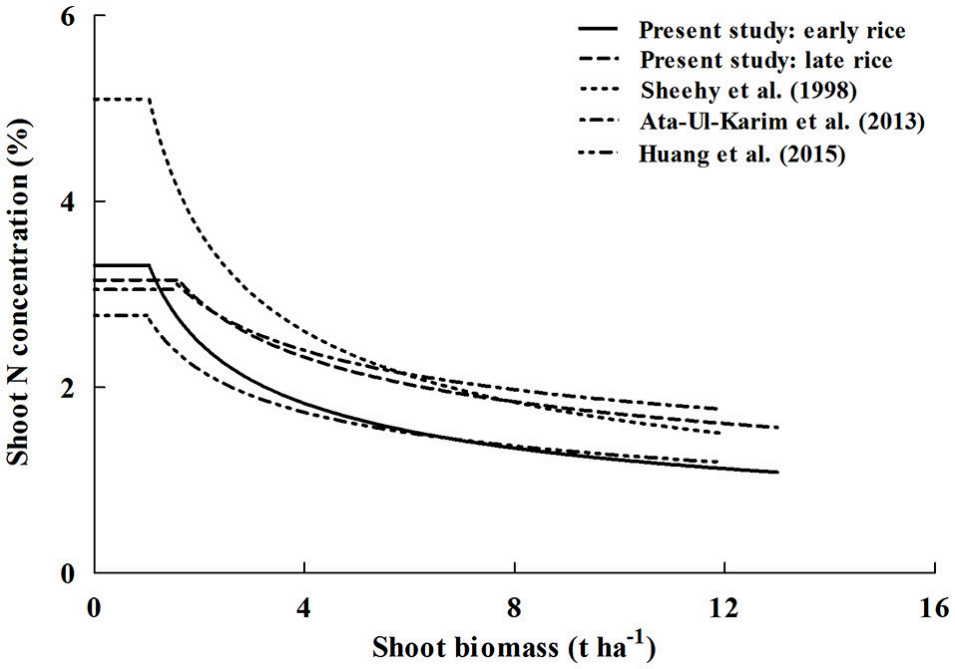
**Comparison of different N_**c**_ dilution curves in rice**. The (____) line represents N_c_ curve of early rice in this study (N_c_ = 3.37 W^−0.44^), the (__ __) line represents N_c_ curve of late rice in this study (N_c_ = 3.69 W^−0.34^), the (……) line represents N_c_ curve of Indica rice of Sheehy et al. (1998) (N_c_ = 5.20 W^−0.45^), the (__ . __ ) line is for Japonica rice in Yangtze River reaches of Ata-Ul-Karim et al. (2013) (Nc = 3.53 W^−0.28^), the (__ . . .) line is for Japonica rice in Heilongjiang (Huang et al., 2015) (N_c_ = 2.77 W^−0.34^).

For Japonica rice dilution curves, parameter *b* is approximately 0.3 and obviously lower than that of early rice and IR72. Parameter *a* from a previous study conducted by Huang et al. (2015) was 2.77, nearly 35% less N accumulation at the early growth stage than Indica rice and Japonica rice of Ata-Ul-Karim et al. (2013) (Figure 7). This may due to the fact that typical Indica varieties and Japonica varieties have significant differences in several factors such as photosynthesis (Ji and Jiao, 2001), metabolism (Hu et al., 2014). Previous studies have shown that plant N content in Indica rice is significantly higher than Japonica rice at the maximum tillering stage (Yoshida et al., 2006). However, early rice N content is particular lower than Japonica rice of Ata-Ul-Karim et al. (2013) due to lower temperatures and solar radiation. And Japonica rice in high latitudes has a slower growth rate than Indica rice owing to genotype and environmental conditions resulting in a slower decrease of N uptake per unit biomass accumulation in Japonica (Ata-Ul-Karim et al., 2013).

### Nitrogen nutrition index and relative yield

Nitrogen nutrition index based on the N_c_ dilution curve is a useful tool to diagnose deficient and non-deficient N nutrition status for crops (Lemaire et al., 2008). The NNI values observed in this study were similar to those obtained for corn (0.3–1.35) (Ziadi et al., 2008), durum wheat (0.25–1.5) (Debaeke et al., 2006) and for rice (0.6–1.05) (Ata-Ul-Karim et al., 2013). NNI values were at a maximum at jointing stage. This is due to N concentration increasing after topdressing at tillering, leading to a high value of NNI, which implies the NNI may cause volatility with fertilizer or change of soil N condition. This result is similar to Lemaire and Gastal (1997).

Nitrogen nutrition index can also be used to assess the effect of N on crop yield (Jeuffroy and Recous, 1999). Higher yields are always associated with high plant N, however, excessive N fertilizer cannot always increase crop yield (Ying et al., 1998). In this study, positive linear correlation between NNI_int_ and RY was estimated at different growth periods. Minor differences between early and late rice imply the NNI_int_ are stable at each period. In Table 3, the RRMSE are under 10% and the accuracies are closed to 1.0 illustrating models of each period have good yield prediction accuracy across different years. Several attempts have been made for in-season estimation of RY on the basis of NNI in spring wheat, corn, sunflower, barley and rice (Ziadi et al., 2008, 2010; Debaeke et al., 2012; Yuan et al., 2016; Zhao et al., 2016b; Ata-Ul-Karim et al., 2016a,b, 2017a,b), but the relationships derived in previous studies were based on instantaneous NNI, either using the averaged NNI data at different crop growth stages or at particular crop growth stage, which may lead to an over or underestimation of grain yield in N deficiency or luxury consumption nutrition conditions (Lemaire and Gastal, 1997). According to Lemaire et al. (2008), using NNI_int_ can avoid the drawback of instantaneous NNI and average values of NNI. Therefore, NNI_int_ may be more reliable for predicting grain yield.

Nitrogen nutrition index can be a good indicator for diagnosing and directing N fertilization during vegetative growth period to improve crop yields. Moreover, the relationship between NNI_int_ and RY further indicates the effectiveness of the Nc dilution curve as a diagnostic tool. NNI values can also be integrated into the growth models such as RiceGrow (Tang et al., 2009) and CERES-Rape (Gabrielle et al., 1998). However, real-time determination of shoot biomass and plant N concentration are the obstacle in directly using the NNI as a pre-diagnosis tool. Modern real-time diagnostic techniques such as satellite imagery (Cohen et al., 2010; Huang et al., 2015), chlorophyll meter (Yuan et al., 2016; Zhao et al., 2016a), and hyperspectral imaging based on UAV (Unmanned Aerial Vehicle) (Pölönen et al., 2006) and ground based canopy reflectance (Liu et al., 2015) can be used to determine plant biomass and plant N concentration non-destructively.

## Conclusions

The shoot biomass increases with growth but at the same stage the increase is not significant with the excess N application treatment. The N concentration gradually declines during the growth period and a higher N application leads to a higher N concentration. The constant N concentration at early growth stage was 3.31 and 3.15% DM for early and late rice, respectively. N_c_ dilution curves based on shoot biomass for double cropping rice were developed (early rice: N_c_ = 3.37 W^−0.44^ and late rice: N_c_ = 3.69 W^−0.34^). Compared with existing reference curves for Indica and Japonica rice, the curves for early and late rice are different from other rice in different climates. The relationships between NNI_int_ and RY established at different crop growth periods showed reliable prediction of yield across years. The NNI calculated from the established N_c_ dilution curves could be used as a reliable indicator for diagnosing crop N status. Results in this study provide a technical support for N fertilization management in the area of rice double cropping in south China.

## Author contributions

ZH and LT wrote the manuscript; ZH, SA, and LT analyzed the experiments data; SA, XQ, YZ, and WC provided advice and edited the manuscript; LT, WC, and YZ planned experiments and ZH, YL, XQ, XL, and QC performed experiments. All authors read and approved the final manuscript.

## Funding

This work was supported by the National High-Tech Research and Development Program of China (2013AA100404), the Special Program for Agriculture Science and Technology from the Ministry of Agriculture in China (201303109), National Science Foundation of China (31571566; 31201130), Jiangsu Agriculture Science and Technology Innovation Fund (CX[14]2116), the Three-new Agriculture Project of Jiangsu Province (SXGC[2014]304), and the Priority Academic Program Development of Jiangsu Higher Education Institutions (PAPD) of China.

### Conflict of interest statement

The authors declare that the research was conducted in the absence of any commercial or financial relationships that could be construed as a potential conflict of interest.

## Footnotes

^1^National Bureau of Statistics of China (2012). Available at http://data.stats.gov.cn/english/easyquery.htm?cn=C01

## Abbreviations

N: nitrogen
N_c_: critical nitrogen
NNI: nitrogen nutrition index
RY: relative yield
NNI_int_: integrated nitrogen nutrition index
N_actual_: actual shoot nitrogen concentration
DM: dry matter
W: dry weight
GY_treatment_: actual yield of each N treatment
GY_max_: the mean of the yield for the group of treatments giving the highest yield value
LSD: least significant difference
GLM: generalized linear model
ANOVA: analysis of variance
ANOCOVA: analysis of covariance
N_max_: nitrogen maximum curve
N_min_: nitrogen minimum curve
UAV: unmanned aerial vehicle
ZJZ-17: zhongjiazao 17
TLY-83: tanliangyou 83
TYHZ: tianyouhuazhan
YY-9113: yueyou 9113
XY-186: xiangyou 186
WFY-788: wufengyou 788.
